# A Species of Elephantiasis from a Scrofulous Cause

**Published:** 1800-07

**Authors:** John Bell

**Affiliations:** Surgeon. His Majesty's Ship, Pelican, Plymouth


### *To the Editors of the Medical and Phyſical Journal.*

GENTLEMEN,

IN looking over Profeſſor HUFELAND's deſervedly celebrated Treatiſe on Scrofula,* I was ſtruck with the following caſe, which he conſiders as an uncommon one, and ſays, that he had never heard of any thing ſimilar, except ſome caſes related by *Hillary* in his *Obſervations on the Diseases, &c. of Barbadoes.* I have been induced to tranſlate it, as I do not know that the original work has yet appeared in an Engliſh dreſs, and hope you will do me the favour to inſert it in your excellent Journal. I have added a caſe, which I had an opportunity of obſerving a few years ago, ſo nearly ſimilar that they may be conſidered as analogous. I am,

GENTLEMEN,

Your obedient ſervant,

JOHN BELL, Surgeon.

*His Majeſty's Ship, Pelican,*

*Plymouth, June 10, 1800.*

---

### " *A Species of Elephantiaſis from a Scrofulous Cauſe.*

" A girl of a ſcrofulous habit, who had frequently running ears, ſore eyes, and other ſymptoms of a ſcrofulous ſtate during her infancy, had paſſed very favourably through the ſmall-pox in her eleventh year, and had enjoyed a good ſtate of health for the two ſucceeding years, was attacked in her thirteenth year with a ſwelling in the left foot, which ſoon extended to the calf of the leg. After ſome general internal and external remedies had been uſed, the tumefaction diſappeared, but returned in a ſhort time, and increaſed to ſuch a degree, that it extended from the toes up to the hip: the leg was more than double the ſize of the other, rather cold than warm, pale, and the ſwelling ſo hard, that no impreſſion could be left on it with the

---

* See *Hufeland Ueber die Natur, Erkenntniſſmittel, und Heilart der Skrofelkrankheit, &c.* 2d edit. *Jena,* 1797.

the finger. The patient was in other refpects apparently healthy, had a blooming hue on her countenance, and a cheerful temper, but had fometimes pain and reftlefsnefs; the natural and vital functions were good.

" After having thus paffed a period of fix months without ufing any medicine, a phyfician was confulted, who treated her chiefly with evacuants and laxatives. From this time fhe became very languid, loft her colour and appetite; the foot got no better, and the idea of curing her being abandoned, fhe in a few months acquired her former ftrength and cheerfulnefs.

" Some time afterwards a veficle appeared on the infide, a little above the knee of the difeafed leg, from which a yellowifh watery fluid was difcharged. This difcharge ceafed fpontaneoufly, but returned in a fhort time, when the matter took on the appearance of curdled milk, and became fo corrofive, as to excoriate the furrounding fkin. The leg became ftill larger and harder, and the fkin in many places felt knotty, and as firm as leather; the other leg remained perfectly found. About this time fhe was attacked occafionally with violent feverifh paroxyfms, attended with anxiety, delirium, and congeftions of blood in the head, which were caufed by the near approach of the natural period of menftruation.

" Some months afterwards, when fhe had attained her fixteenth year, and the difeafe had been of three years ftanding, fhe was ordered to take *fpongia ufta*, *radix ferpentariæ*, *pilulæ fcillæ*, and *decoctum ligni guiaici*; fomentations of herbs, with *camphor* and *volatile alcali*, were applied externally to the leg, and *aqua lytharg. acet. comp.* to the fore.

These medicines caufed diarrhœa, and fometimes vomiting, and increafed the urinary difcharge, but the leg got no better, except that the fore was dried up: the medicines were continued. A few weeks afterwards fhe had another attack of the feverifhnefs mentioned above, with anxiety, delirium, and difordered circulation, which continued for fome days; and immediately afterwards, the difeafed leg was fomewhat fofter, and acquired a red and purplifh colour. A fhining and very tenfe tumour arofe under the calf, in which the patient complained that there was a violent and painful pulfation.

" She continued the fame medicines, with the addition of Hoffman's fublimate pills. A fortnight afterwards the tumour under the calf of the leg inflamed greatly, burft, and difcharged a yellow-coloured pus, and the fore above the knee alfo broke out again. In a little time the leg became fomewhat fmaller.

" For a whole year after this, the moft active medicines, as *hydrarg. muriat. pulvis plummeri*, *arnica*, *assafœtida*, *zinc. vitriolat*, *cicuta*, friction with *ungt. hydrarg. fort.* fumigations with with *hydrarg. sulphurat. rub.* warm baths and ſhower baths, with chalybeate and ſulphureous waters, were made uſe of.

" The ſize and hardneſs of the leg were not diminiſhed by theſe means, but rather ſeemed to become more conſiderable. Beſide the ſores already mentioned, ſeveral hard glandular tumours appeared, which inflamed, ſuppurated, and diſcharged an acrid lymph, which often cauſed great pain in the ſurrounding parts. She had feveriſh attacks from time to time, which continued a few days, and generally left the leg much worſe. Her menſes appeared in the middle of the year, without in the leaſt changing this dreadful evil.

" At the cloſe of the year, the leg was of a monſtrous circumference, felt hard and ſcirrhous quite up to the hip, and was in many parts covered with ſcabbed, ſuppurating, and inflamed places. What is very remarkable, the growth of the patient went on; her menſes were regular, ſhe ate and drank with an appetite, and all her functions were natural. The diſeaſe remained entirely topical.

" In this way ſhe remained for three years longer, during which the diſeaſe ſtill increaſed, and died at laſt from hectic fever and atrophy.

" I take this, which is certainly a very remarkable caſe, from the papers of my late revered father. From every ſymptom, and from the whole tenour of the diſeaſe, it is evident that it was nothing more than a diſeaſe of the glands and lymphatic veſſels of the part, and therefore a ſcrofulous diſeaſe; and hence it is apparent to what a dreadful height it may arrive, and likewiſe for what a length of time it may remain merely topical.

" With reſpect to the appearance which the diſeaſe here took on, I have read of nothing ſimilar, except the caſes which Hillary deſcribes under the appellation of Elephantiaſis, the diſtinguiſhing ſymptoms of which were a monſtrous hard glandular ſwelling of one leg, a totally indurated cellular membrane, and a rough, irregular, and ſcaly ſurface, with which the patient often lived, for twenty years, without any other function being diſturbed by it. He alſo remarks feveriſh attacks from time to time, which invariably increaſed the evil, and threw freſh metaſtaſes on the diſeaſed leg."

---

A girl of thirteen years of age was ſeized with general ſwelling of the left leg, from the knee to the ancle; and it was ſo painful that ſhe could not bear to have it touched. It looked a little red for a few days, and afterwards became pale; but without any diminution of pain, though the ſwelling rather decreaſed.

creafed. A week afterwards fhe was attacked with feverifh-nefs, thirft, reftlefsnefs, and great heat of the fkin, for which fome laxative medicines and antimonials, with opiates at night, were ordered; and the leg was rubbed, whenever fhe could be prevailed upon to fubmit to it, with *ungt. ceræ* and *calomel.* About a month after the firft attack, the pain became more violent; fhe could not be prevailed on to lay down in bed, but conftantly fat in an elbow chair, with the foot refting upon a pillow on the floor. She could not now bear to have it rubbed, or even to wear a loofe ftocking or bandage, and was afraid to let her petticoats touch it. In this way it continued about fix months with little variation, except that the whole leg became œdematous and greatly fwelled, fo as to be of more than three times its natural fize.

The fkin and cellular membrane foon became indurated from below the knee down to the toes, poffeffed very little feeling, and felt hard and firm like leather; yet ftill fhe complained of violent deep-feated pain, and fcreamed out in great agony if any one attempted to move her. At the end of a year, feveral fcirrhous-like tumours, of the fize of a filbert, appeared on different parts of the leg and foot, which inflamed, fuppurated, and difcharged a thin, curdled ferum; they were not very painful.

In this way fhe continued for another year, the leg increafing in thicknefs, and the fkin and cellular fubftance growing ftill harder; and during this period fhe was fubject to frequent feverifh attacks, which reduced her ftrength greatly, and wore her down to a mere fkeleton.

Towards the clofe of the year, an abfcefs, of confiderable fize, appeared on the *trochanter major* of the left thigh, which burft, and difcharged at firft a large quantity of good pus, which was foon changed to a thin ferum; and after having been ill above two years and a half, fhe died during one of the feverifh attacks, already mentioned, without ever having lain down in bed during that period, after the firft month.

It was thought from the firft, and during the progrefs of the difeafe, to be a cafe of *Necrosis Ossium*; but on examining after death, no fuch difeafe was found, for the bones of the leg and foot were found, but rather fofter than ufual. The adipofe membrane, which contained a great quantity of ferum in its cells, and the fkin, were found to be the fole feat of the difeafe.

A variety of medicines were tried in this cafe, but without the leaft good effect, and fhe only experienced relief from large dofes of opium.

